# Blue Glass Cure

**Published:** 1877-05-20

**Authors:** 


BLUE GLASS CURE.—We notice that the editor of the *Boston Journal of Chemistry* and other chemical writers, place little confidence in the wonderful properties ascribed to blue glass as a therapeutic agent.

